# Ileal pouch of ulcerative colitis and familial adenomatous polyposis patients exhibit modulation of autophagy markers

**DOI:** 10.1038/s41598-018-20938-5

**Published:** 2018-02-08

**Authors:** Nielce Maria Paiva, Lívia Bitencourt Pascoal, Leandro Minatel Vidal Negreiros, Mariana Portovedo, Andressa Coope, Maria de Lourdes Setsuko Ayrizono, Claudio Saddy Rodrigues Coy, Marciane Milanski, Raquel Franco Leal

**Affiliations:** 1 IBD Research Laboratory, Coloproctology Unit, Surgery Department University of Campinas (UNICAMP), Medical School, Sao Paulo, Brazil; 2 Laboratory of Metabolic Disorders, Faculty of Applied Sciences University of Campinas (UNICAMP), Sao Paulo, Brazil

## Abstract

Total retocolectomy with ileal pouch-anal anastomosis (IPAA) is the surgery of choice for patients with ulcerative colitis (UC) that are refractory to clinical treatment. Pouchitis is one of the most common complications after this procedure. Defects in autophagy have been reported in inflammatory bowel diseases. However, there are no studies on the IP. Therefore, we studied markers for autophagy in the IP mucosa of UC and FAP patients comparing them to controls with a normal distal ileum. Sixteen patients with IP in “J” shape, asymptomatic and with endoscopically normal IP were evaluated. The control group consisted of eight patients with normal colonoscopy. There was a significant decrease in the transcriptional levels of *ATG5, MAP1LC3A* and *BAX* in the FAP group. There was also a decrease in the protein level of Beclin-1 in the UC and FAP compared to the control group. Although the LC3II levels by immunoblot were higher in the UC group, LC3/p62 co-localization were lower in the immunofluorescence analysis in the UC and FAP compared to the control group. Corroborating these results, there was an increase of p62 by immunoblot in the UC group. These findings indicated a modulation of macroautophagy markers in the IP, which may explain the mucosa inflammation predisposition.

## Introduction

Ulcerative colitis (UC) is a chronic intestinal inflammation that can affect the large intestine and rectum. Its etiology is not completely established. Familial adenomatous polyposis (FAP) is an autosomal dominant disease which affects young individuals and is associated with the formation of multiple polyps in the large intestine and rectum, which invariably implies a greater risk of cancer^1,2^. Both diseases, despite being different, may require the same surgical procedure. The ileal pouch-anal anastomosis (IPAA) is the elective procedure of choice in the surgical management of refractory UC, and FAP with many polyps in the rectum^3^. The main complication after this procedure is the pouch inflammation (pouchitis) that can affect up to 45 percent of patients who are submitted to IPAA for UC, and only five percent of the FAP patients who undergo the same procedure^4^. This suggests that constitutive differences between UC and FAP pouches have a critical role in its pathogenesis.

Pouchitis develops only after ileostomy closure, when the pouch mucosa starts to be exposed to the fecal stream^5^. The distinct immunological aspects of the different inflammatory bowel diseases (IBD), specifically UC, which involve impaired innate and adaptive responses, associated to genetic susceptibility, environmental factors, and intestinal microbiota may be involved in the pouch inflammation etiology^5,6^.

Autophagy is an evolutionarily conserved catabolic pathway that consists of selective degradation of cellular components and a homeostatic mechanism that protects cells exposed to stress situations (toxins, starvation)^7,8^. There are three primary forms of authophagy: macroautophagy, microautophagy and chaperone-mediated autophagy (CMA)^9^. Although there are no indications of genetic mutations related to the mechanism of autophagy associated to UC susceptibility, the transcriptional and protein evaluation of this mechanism in the ileal pouch mucosa is of fundamental relevance. Alterations of apoptosis in this tissue have already been described previously^10^ and both signaling pathways, autophagy and apoptosis, are interconnected^11,12^. Indeed, differential expression of Beclin1, a relevant protein that is involved in the initiation of autophagy, was already seen in the colon of UC patients^4^. Moreover, epigenetic alterations, which have recently been related to the etiology of IBD^13,14^, can determine transcriptional changes, which in turn help to better understand the mechanisms that predispose patients to the inflammatory process in the ileal pouch justifying its study. Thus, we evaluated molecules involved in the autophagy pathways in ileal pouch mucosa of UC and FAP patients, even in the absence of clinical, endoscopic and histological inflammation, in order to understand if there is underlying modulation in these pathways that could mediate molecular inflammation in the IP.

## Results

### Histological analysis to evaluate the Pouchitis Disease Activity Index

**Figure 1:**
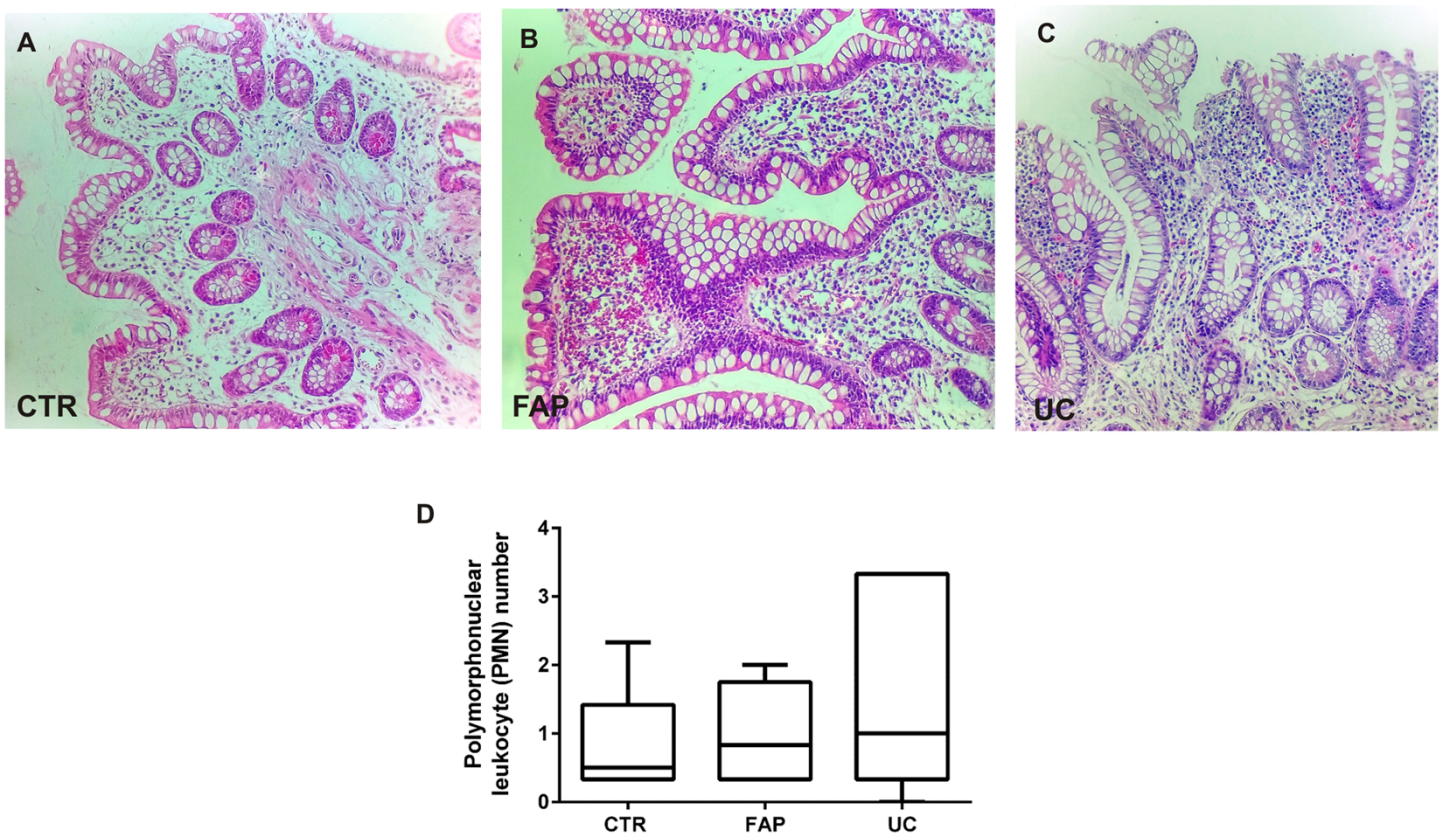
Haematoxylin and Eosin (H&E) staining of ileal pouch mucosa biopsy of representatives Familial Adenomatous Polyposis (FAP) and Ulcerative Colitis (UC) patients. (**A**) Ileal pouch mucosa of a normal control (CTR Group). (**B**) Ileal pouch mucosa of FAP patient (FAP Group). (**C**) Ileal pouch mucosa of UC patient (UC Group). (**D**) Polymorphonuclear (PMN) leukocyte number of the *lamina propria* in the CTR, FAP and UC Groups. There is no statistical difference among the groups. Nuclear counterstaining: Mayer’s haematoxylin. Original magnification X20.

Among the aspects analyzed by the PDAI, the histological analysis of the biopsies collected in the ileal pouch mucosa of UC and FAP patients demonstrated a small amount of acute inflammatory cells infiltration, absence of crypts destruction, cell architecture preservation and presence of goblet cells, as demonstrated in Fig. 1B,C. Similarly, no significant histological changes were found in the biopsies performed in normal ileum (Control group) (Fig. 1A).

The polymorphonuclear leukocyte count of the *lamina propria* is shown in the Fig. 1D. Photomicrographs were taken using a Leica DM 4500B microscope and Leica DFC 290 digital camera system with Leica Application Suite version 3.8 Software (Leica Microsystems, Wetzlar). Three fields for each sample were captured. The immune cells of the *lamina propria* were counted for quantitative analysis, which was analyzed by two blinded observers (N.M.P. and L.B.P.) in a panchromatic objective field of higher magnification 40X.

PDAI was performed for all patients taking into account clinical, endoscopic and histological aspects (see supplementary figure). All patients evaluated had PDAI < 7. The aim of our study was to analyze patients without inflammation to show if there were underlying molecular alterations in the ileal pouch mucosa, even in the absence of endoscopic and histological inflammation.

### Transcriptional analysis of the autophagy related genes in the ileal pouches from fap and uc patients

**Figure 2:**
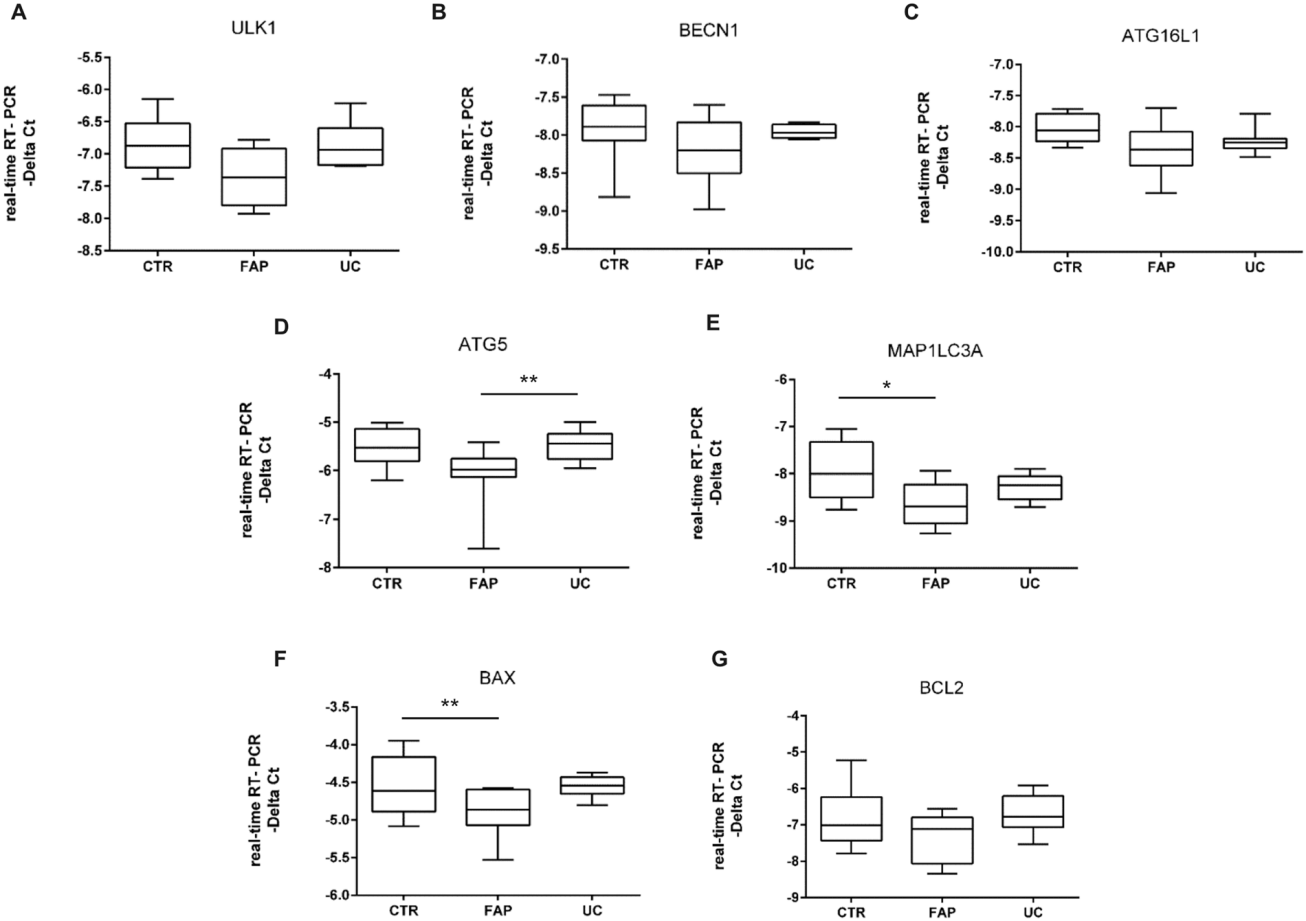
Evaluation of autophagy and apoptosis related gene expressions in the ileal pouch mucosa of Familial Adenomatous Polyposis (FAP) and Ulcerative Colitis (UC) patients. Transcriptional analysis reveals autophagy markers modulation in the ileal pouch mucosa of FAP patients. mRNA levels (qRT-PCR) of *ULK1* (**A**), *BECN1* (**B**), *ATG16L1* (**C**), *ATG5* (**D**), *MAP1LC3A* (**E**), *BAX* (**F**) and *BCL2* (**G**) in ileal pouch mucosa of controls (CTR Group), FAP patients (FAP Group) and UC patients (UC Group). For FAP, n = 8; for UC, n = 8; for CTR, n = 8; *p < 0.05, **p < 0.01 and ***p < 0.001.

Patients with FAP showed decreased mRNA levels of *ATG5* in the ileal pouch when compared to UC (p < 0.01; Fig. 2D), and decreased levels of *MAP1LC3A* compared to the controls (p < 0.05; Fig. 2E). No differences were observed in the other genes (p > 0.05; Fig. 2A,B and C). To explain these findings, we decided to evaluate apoptosis related genes and although there was no statistical differences in *BCL2* expression, an anti-apoptotic gene (p > 0.05; Fig. 2G), we found decreased *BAX* levels in the FAP group when compared to CTR group (p < 0.01; Fig. 2F). *BAX* encodes a pro-apoptotic protein, that when it is decreased, it leads to the inhibition of autophagy related genes, such as *ATG5*.

### Transcriptional analysis of the autophagy related genes in the afferent limb of ileal pouches from fap and uc patients

**Figure 3:**
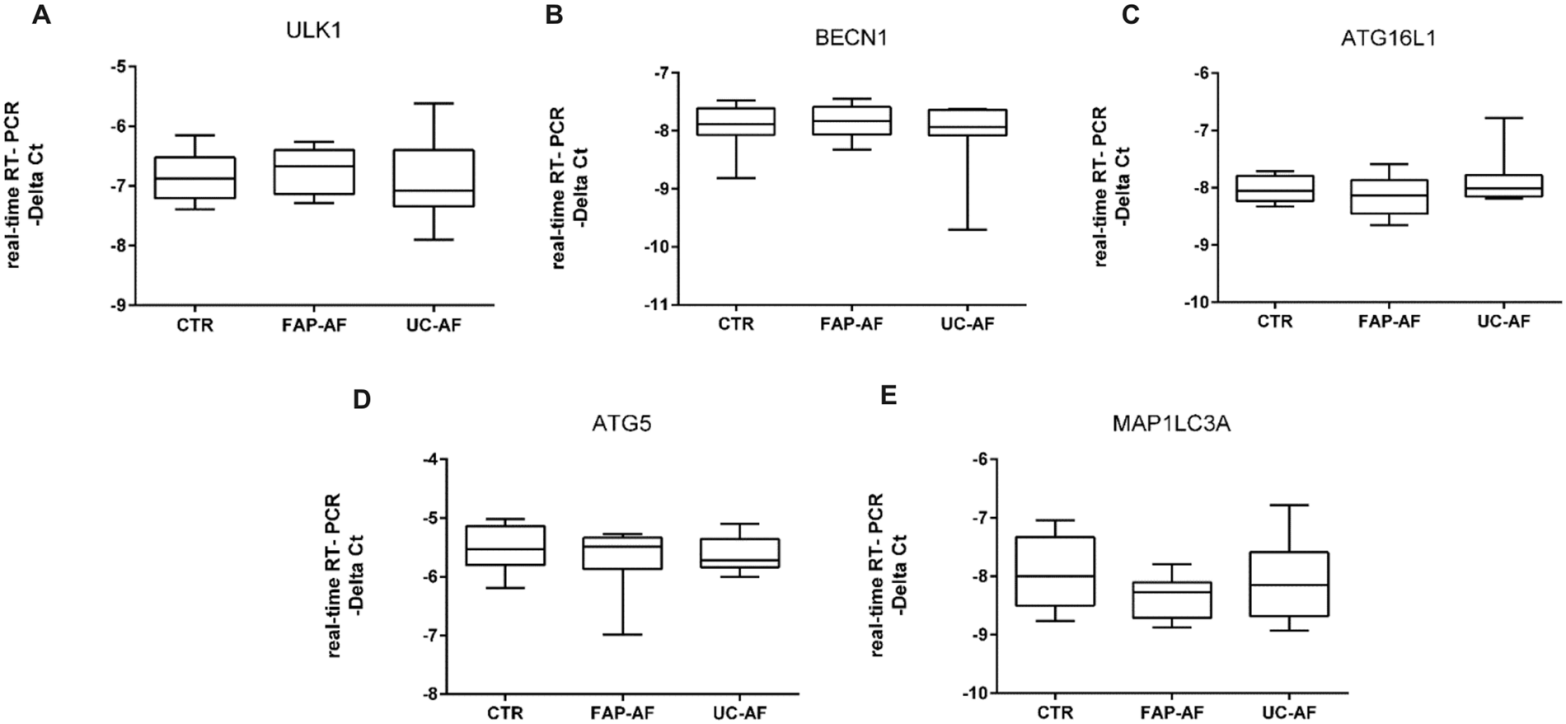
Evaluation of autophagy gene expressions in the ileal pouch afferent limb mucosa of Familial Adenomatous Polyposis (FAP) and Ulcerative Colitis (UC) patients. Transcriptional analysis reveals no differences compared to the controls. mRNA levels (qRT-PCR) of *ULK1* (**A**), *BECN1* (**B**), *ATG16L1* (**C**), *ATG5* (**D**), and *MAP1LC3A* (**E**) in ileal pouch afferent limb mucosa of controls (CTR Group), FAP patients (FAP-AF Group) and UC patients (UC-AF Group). For FAP-AF, n = 8; for UC-AF, n = 8; for CTR, n = 8; *p < 0.05, **p < 0.01 and ***p < 0.001.

There were no differences in the mRNA levels of autophagy related genes comparing the control, FAP-AF and UC-AF groups (p > 0.05; Fig. 3), which shows no transcriptional alterations among the afferent limbs and the normal terminal ileum mucosa.

### Protein analysis by immunoblotting revealed modulation of autophagy markers in the ileal pouch of uc and fap patients

**Figure 4:**
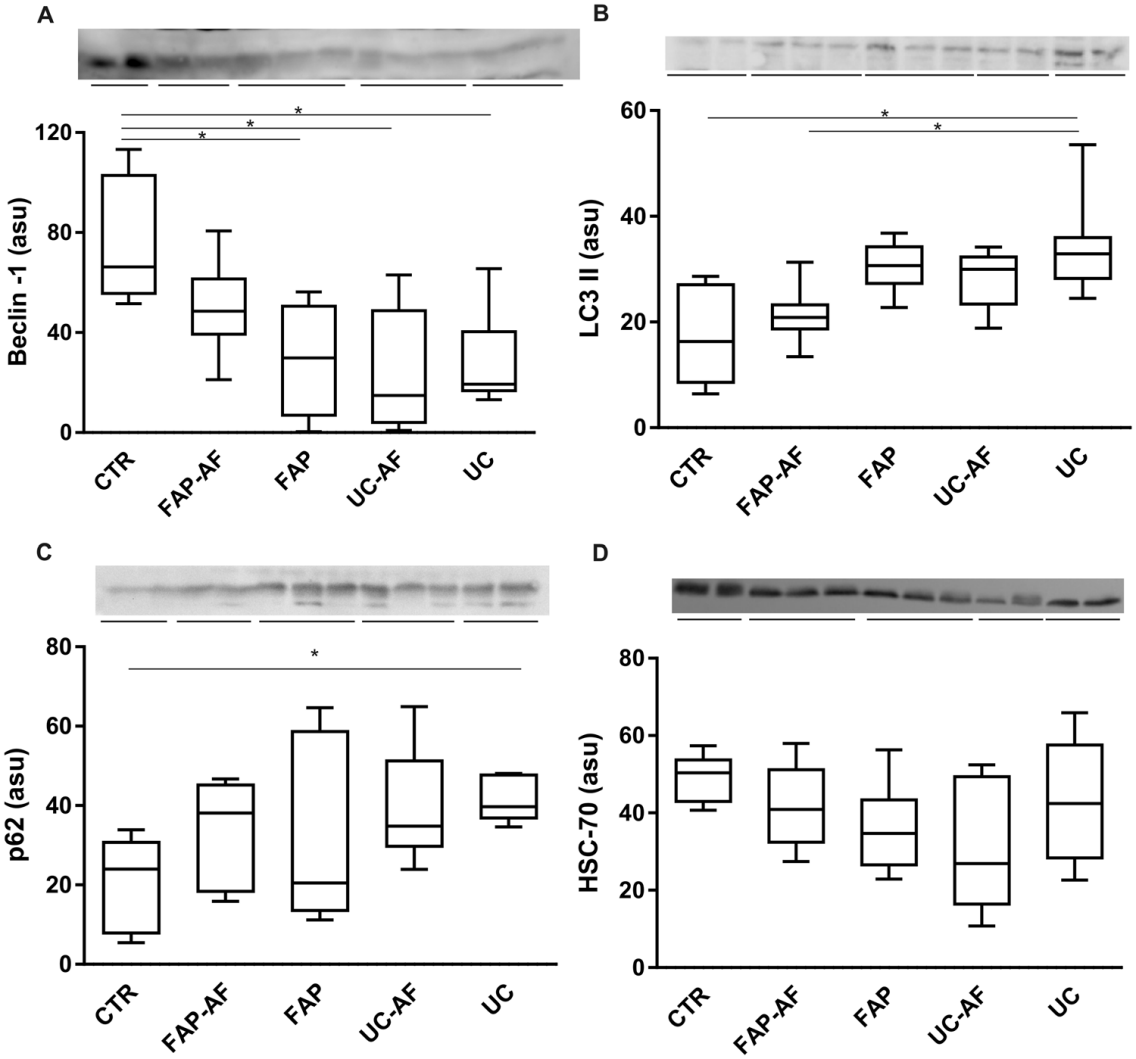
Ileal pouch mucosa of Familial Adenomatous Polyposis (FAP) and Ulcerative Colitis (UC) patients shows autophagy protein markers modulation. Western blot analysis of Beclin-1 (**A**), LC3 (**B**), p62 (**C**) and HSC-70 (**D**) in ileal pouch (FAP and UC Groups) and in its afferent limb mucosa (FAP-AF and UC-AF Groups) of FAP and UC patients compared to controls (CTR Group). Each band represents one patient. For FAP, n = 8; for UC, n = 8; for CTR, n = 8; for FAP-AF, n = 8; for UC-AF, n = 8; *p < 0.05, **p < 0.01 and ***p < 0.001. ASU: arbitrary scanning unit.

In order to better evaluate the autophagy pathway, we measured the proteins amount by immunoblotting using the same samples of PCR analysis. We found decreased levels of Beclin-1 in FAP, UC-AF and UC groups when compared to control group (p < 0.05; Fig. 4A). Although LC3 level was increased in UC patients when compared to CTR and FAP-AF groups (p < 0.05; Fig. 4B), an increased non-degraded p62 was observed in the UC group (p < 0.05; Fig. 4C). Beclin-1 participates in the early stages of the autophagy pathway. However, p62 is a relevant molecule, which binds to LC3 and is responsible for carrying ubiquitinated unfolded proteins into the autophagosome and enable their degradation in the lysosome. p62 is an adapter protein, thus, if it is increased means failure in the degradation process, i.e. macroautophagy failure.

HSC-70 levels, which is a marker of chaperone-mediated autophagy, were similar among the groups (p > 0.05; Fig. 4D).

### Immunofluorescence protein analysis confirmed modulation of autophagy markers in the ileal pouch of uc and fap patients

To confirm the protein-related autophagy expression data, co-localization for LC3 and p62 was performed.

**Figure 5:**
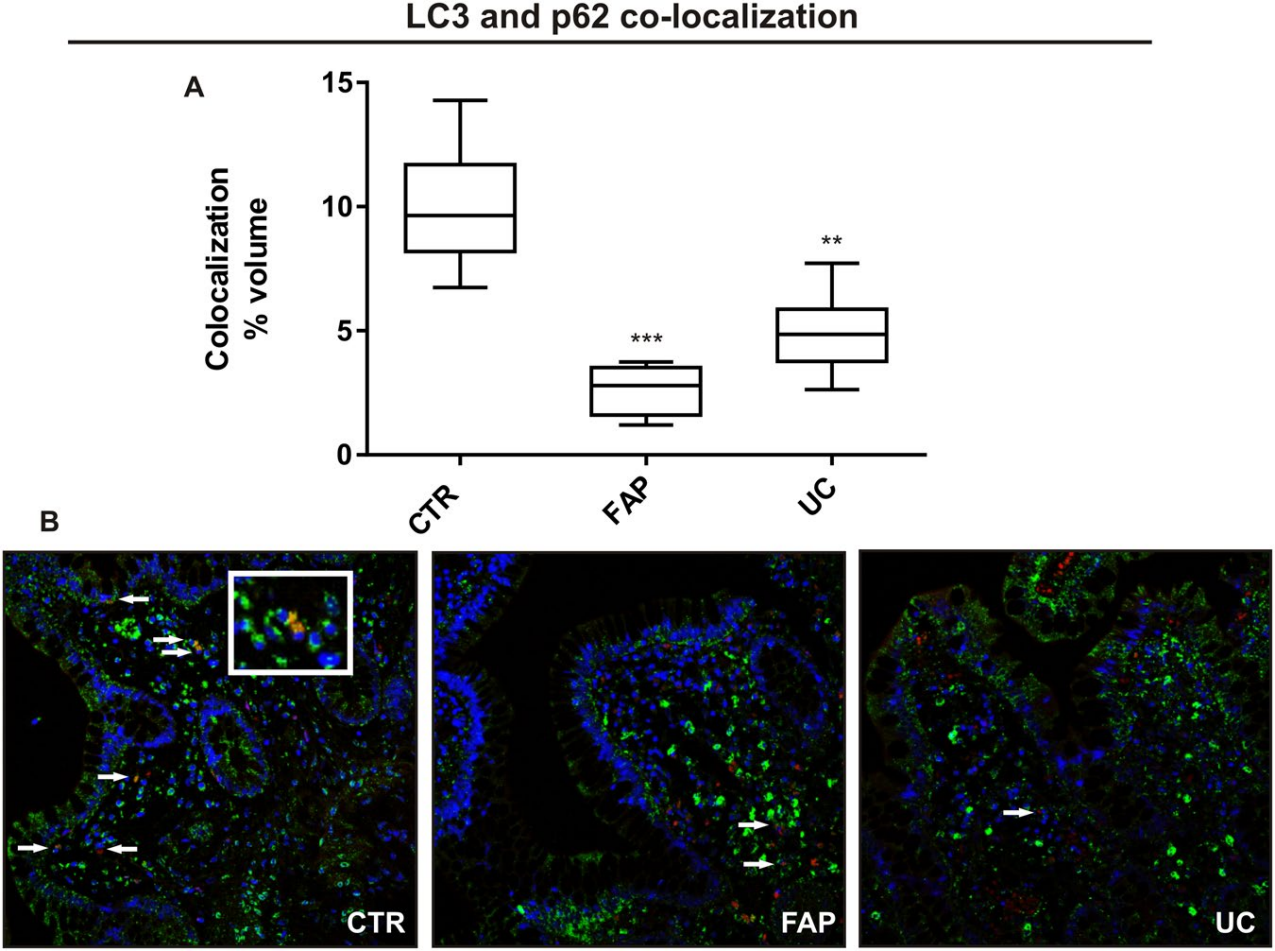
Immunofluorescence staining of LC3 and p62 co-localization in the ileal pouch mucosa of Familial Adenomatous Polyposis (FAP) and Ulcerative Colitis (UC) patients. (**A**) Quantitative analysis of immunofluorescence staining for LC3 and p62 co-localized in FAP, UC and control (CTR) groups. (**B**) Representative staining of fixed paraffin-embedded ileal mucosa from the CTR, FAP and UC groups, showing low number of positive cells in FAP and UC groups compared to the CTR group. Positive cells are shown in orange (co-labeled by PI and FITC; overlay image) or red and green in the same cytosol (co-labeled by Alexa Fluor® 488 and Cy3®). Nucleus was stained with DAPI (blue-fluorescent). The arrows show the positive cells. Images were obtained using a 40X objective. For FAP, n = 8; for UC, n = 8; for CTR, n = 8; *p < 0.05, **p < 0.01 and ***p < 0.001.

Despite the increased LC3 II levels verified in the ileal pouch mucosa of UC patients by immunoblotting, the immunofluorescence analysis revealed a significantly lower number of LC3 and p62 co-localized cells in the FAP and UC groups, when compared to the CTR group (p < 0.05; Fig. 5A, quantitative analysis). Representative images of LC3 and p62 co-staining in the ileal pouch mucosa of normal distal ileum, FAP and UC patients are shown in Fig. 5B, where the positive cells are well identified in the different groups (orange/yellow in the cell cytoplasm; nucleus is counter-stained in blue).

## Discussion

Previous studies have shown increased pro-inflammatory cytokines, nuclear transcription factor STAT-1, and bacterial antigen receptors such as TLR4 in the ileal pouch mucosa of UC patients, when compared to FAP and controls of normal distal ileum, even in patients without pouchitis^15–17^. These findings showed the greater susceptibility of UC patients to the inflammatory process in the ileal pouch after IPAA. In addition to those findings, a decrease in apoptosis in the ileal pouch mucosa of UC patients was also reported in the literature^10^. It is known that there is a close relationship between the mechanism of apoptosis and autophagy^7,9,11^. Hao X *et al*. verified increased levels of beclin-1 in colonic mucosa of UC patients^18^. However, there were no studies evaluating cellular autophagy, a relevant mechanism for recycling dysfunctional cellular components present in the cytoplasm, in the ileal pouch of UC and FAP patients.

There are three primary forms of authophagy: macroautophagy, microautophagy and chaperone-mediated autophagy (CMA)^7–9,19^. In macroautophagy, targeted cytoplasmic components are isolated from the rest of the cell within a double-membraned vesicle (autophagosome)^20,21^. The autophagosome can fuse with lysosomes and the proteins are degraded and recycled. On the other hand, microautophagy is mediated by direct lysosomal engulfment of the cytoplasmic components, which is trapped in the lysosome by the membrane invagination^22^. The recognition of the protein substrate by specific proteins such as chaperones in the cytosol, binding directly to the lysosome, translocating across it, without additional vesicles formation, is the characteristic of CMA^23^. For this reason, autophagy prevents the accumulation of abnormal proteins, is also involved in the genomic stability, and participates of the removal of intracellular pathogens^24^. When there is an autophagy deficiency, it promotes cytoplasmic protein inclusions, which are composed of misfolded proteins. The accumulation of deformed organelles can lead to cell injury and diseases^8,25^.

In the present study, the purpose was to study patients without pouchitis to demonstrate if there was underlying modulation of autophagy markers in the ileal pouch mucosa, even in the absence of endoscopic and histological inflammation. For this, we applied the PDAI (Pouchitis Disease Activity Index)^26^, and all patients had PDAI less than seven points. We showed increased LC3 II levels in the ileal pouch mucosa of UC patients by immunoblotting analysis. However, the decreased levels of beclin-1 and non-degraded p62 observed in Fig. 4A,C respectively, and also the less number of LC3/p62 co-localized cells in the UC group compared to controls reinforce the finding of autophagy markers defective modulation. Beclin-1 initiates the autophagy process. LC3 enroll in the autophagossome formation and p62 binds to LC3 and is responsible for carrying abnormal proteins into the autophagosome^27^. When p62 is accumulated in the cytoplasm, not binding to LC3, it signalizes that the macroautophagy may be deficient, even higher LC3 II levels is detected. Additionally, we did not verify altered CMA markers between the groups.

An interesting finding was also the detection of decreased autophagy markers in the ileal pouch mucosa of FAP patients when compared to controls. The transcriptional analysis showed fewer levels of *ATG5* and *MAP1LC3A* in FAP group, besides decreased Beclin-1 protein levels by immunoblotting analysis, and finally, decreased number of total LC3 and LC3/p62 co-localized cells verified by immunofluorescence compared to control group. To explain these findings, we decided to evaluate apoptosis related genes. Although there were no statistical differences in *BCL2* expression, we found decreased *BAX* level, which encodes a pro-apoptotic protein, in the FAP group when compared to controls. Decreased apoptosis markers were already described in the ileal pouch mucosa of FAP patients, what may explain the tendency to low cell turn over and possible development of adenomas in this syndrome^10^. The decreased *BAX* levels in FAP group can lead to inhibition of autophagy related genes, as *ATG5*, and may explain the decreased levels of proteins related to autophagy, as we showed in Figs 4 and 5.

Conversely, the defect autophagy in UC pouches may be explained by other mechanism. Increased levels of TLR4 were already observed in UC ileal pouch mucosa, even in the absence of endoscopic inflammation^17^. The relationship between TLR4 and immune system cells, mainly macrophages, frequently are associated to increased autophagy in those cells, especially after LPS treatment, which is a TLR4 agonist^28,29^. However, there is data from animal experimentation that addresses the role of autophagy in macrophage polarization^30^. Despite being in the context of obesity, it shows that in the obese animal, which exhibits increased TLR expression, autophagy is decreased in macrophages isolated from the peritoneum. They correlate this impaired autophagy with changes in the m1 and m2 macrophages profile, leading to inflammation. Therefore, in chronic stimuli such as obesity, autophagy is modulated in some tissues (hypothalamus, liver, muscle and macrophage)^31–35^. There are still no studies in intestinal mucosa addressing the negative regulation of autophagy through TLR activation, which may explain our results in UC ileal pouch mucosa. The mechanisms by which this may happen are still grounds for investigation in other tissues^36^. In fact, we verified decreased of macroautophagy markers in the ileal pouch mucosa of both, UC and FAP, but the mechanisms to explain may be distinct, analyzing data already published. In FAP, decreased autophagy markers may be related to impaired apoptosis, otherwise in UC, may be mainly due to increased TLR activation.

Therefore, autophagy is relevant to the cell survive, since the accumulation of unfolded and abnormal proteins leads to activation of pro-inflammatory pathways. Those evidences of autophagy markers modulation may explain the prone to inflammation in the ileal pouch mucosa, mainly in UC. However, some limitations should be considered. First, this is a descriptive cross-sectional observational study and did not intend to correlate with the occurrence of future clinical manifestations. For this purpose, we would need a longitudinal study with a larger cohort and long-term follow-up. Second, we did not measure the autophagic flux directly. However, the ubiquitin-associated protein p62, which binds to LC3, was used to monitor autophagic flux indirectly, as we did in Fig. 5. In addition, we applied several assays to confirm the results, and also to explain contradictory ones. These findings were the first to show modulation of autophagy markers in the ileal pouch of UC and FAP, even in patients without clinical and endoscopic inflammation.

This subject deserves further studies and detailed mechanisms^37,38^, which can help to find out new targets to ameliorate inflammation in the ileal pouch and even in UC. If these findings are confirmed in a longitudinal study, exploring the correlations with clinical settings, then this work provides novel insight into the complex pathogenesis of primary pouch inflammation.

## Methods

Mucosal biopsies were obtained from eight patients with non-inflamed IPAA after rectocolectomy for UC [median age, 52 (range, 38–66) years; 75% male; 25% female], and eight patients with non-inflamed IPAA after rectocolectomy for FAP [median age, 52.5 (range, 35–70) years; 37.5% male; 62.5% female]. Biopsies of the intestinal mucosa of these patients were collected from the ileal pouch (UC and FAP Groups) and from the afferent limb of the ileal pouch (UC-AF and FAP-AF Groups). The postoperative follow-up was 186.5 (13–360) months. The reservoir design was of the “J” type, and the right colon vascular arcade was preserved as a supplementary blood supply to the terminal ileum^39^. All the patients in this study had the absence of pouchitis, which was defined clinically, histology and endoscopically, according to the PDAI (Pouchitis Disease Activity Index)^26^ and the ileostomy closed for more than one year. In the control group (CTR Group), eight individuals with normal colonoscopy examination were included, with a median age of 62.5 (range, 53–72) years and 37.5% were female. Six biopsies from each patient were obtained from the terminal ileum (control), from the afferent limb and from the ileal pouch (UC and FAP).

This study was approved by the Ethics Committee of the University of Campinas (UNICAMP), all patient signed the informed consent form, and was performed in accordance with the Declaration of Helsinki. The study was carried out at the University of Campinas, IBD Research Laboratory of the Surgery Department, and at the Laboratory of Cell Signaling of the Internal Medicine Department.

### Histological analysis (hematoxylin - eosin)

Biopsies were embedded in paraffin blocks for histological analysis. Sections of 5 μm were cut and stained with hematoxylin and eosin dye. Photomicrographs were taken using a Zeiss Axiophot microscope and Cannon Power Shot G5 digital camera system (Cannon Inc., Tokyo). Fields of higher magnification (20X) were scanned and random fields were analysed. The histological part of the PDAI was performed.

### RT-PCR Analysis

Biopsies from the mucosa of the terminal ileum and from the UC and FAP patients (afferent limb and ileal pouch) were snap-frozen in liquid nitrogen and stored at −80 °C until use. Total RNA was extracted using Trizol (Invitrogen), according to the manufacturer’s instructions. RNA purity and concentration were determined by UV spectrophotometry at 260 nm. RNA was reverse transcribed using oligo (dT) primers and reverse transcriptase (High-Capacity cDNA Reverse Transcription™ Kit, Applied Biosystems). The reaction mixture (20 µl) was incubated at 42 °C for 60 min, then for 10 min at 70 °C, and cooled on ice. RT-PCR was performed on resulting cDNA, using the manufacturer’s protocol, in a 25 µl reaction volume per capillary. Gene-specific primers (TaqManTM - Applied Biosystems™) were *ATG16L1* (Hs00250530_m1), *MAP1LC3A* (Hs00261291_m1), *BECN-1* (Hs00186838_m1), *ATG5* (Hs00169468_m1), *ULK1* (Hs00177504_m1), *BCL2* (Hs00608023_m1), *BAX* (Hs00180269_m1) and *GAPDH* (NM_002046.3). RT-PCR amplification consisted of an initial denaturation step (50 °C for 2 min and 95 °C for 10 min), 40 cycles of denaturation (95 °C for 15 s), annealing (53 °C for 20 s) and extension (72 °C for 20 s), followed by a final incubation at 60 °C for 1 min. All measurements were normalized by the expression of *GAPDH* gene, considered as a stable housekeeping gene^40^.

Real-time PCR analysis of gene expression was performed in a STEP ONE^TM^ Real-Time PCR System (Applied Biosystems). The optimal concentration of cDNA and primers, as well as the maximum efficiency of amplification, were obtained by five-point, two-fold dilution curve analysis for each gene. Real-time data were analyzed using the Sequence Detector System 1.7 (Applied Biosystems). Reagents for Real-time PCR analysis were from Invitrogen (Carlsbad, CA, USA) and Applied Biosystems (Foster City, CA, USA).

### Immunoblotting – Gel electrophoresis

Biopsies were snap-frozen in liquid nitrogen and stored at −80 °C until use. For total protein extract preparation, the fragments were homogenized in solubilizing buffer at 4 °C [1% Triton X-100, 100 mM Tris-HCl (pH 7.4), 100 mM sodium pyrophosphate, 100 mM sodium fluoride, 10 mM EDTA, 10 mM sodium orthovanadate, 2.0 mM phenylmethylsulfonyl fluoride (PMSF), and 0.1 mg aprotinin/ml] with a Polytron PTA 20 S generator (model PT 10/35; Brinkmann Instruments, Westbury, NY) operated at maximum speed for 30 sec. Insoluble material was removed by centrifugation (12000 rpm at 4 °C for 40 min). The protein concentration of the supernatants was determined by BCA method (Pierce^TM^ BCA Protein Assay Kit. Catalog number 23225). Aliquots of the supernatants containing 50 μg total proteins were separated by SDS-PAGE, transferred to nitrocellulose membranes and blotted with indicated antibodies as described in the results. Specific bands were labeled by a chemiluminescence reaction (SuperSignal West Pico Chemiluminescent Substrate from Pierce Biothecnology, Inc. Rockford, IL) and quantified by optical densitometry (Un-Scan-It Program). We have applied Ponceau staining to check equal loading of gels and membrane transfer (see supplementary information)^41,42^.

All the reagents for SDS-polyacrylamide gel electrophoresis and immunoblotting were from Bio-Rad Laboratories (Richmond, CA, USA). HEPES, phenylmethylsulfonyl fluoride, aprotinin, dithiothreitol, Triton X-100, Tween 20, glycerol, and BSA (fraction V) were purchased from Sigma Chemical Co. (St. Louis, MO, USA). Nitrocellulose paper (BA85, 0.2μm) and the reagents for chemoluminescence protein labeling in immunoblots were purchased from Amersham (Aylesbury, UK). The primary antibodies against Beclin (ab-16998), p62 (ab56416 or ab91526) were from AbCam, Cambridge, MA, USA; and LC3 (#2775) from Cell Signaling, Boston, MA, USA or LC3 (ab48394) from AbCam, Cambridge, MA, USA, and Hsc70 (sc7298) from Santa Cruz Biotechnology, Santa Cruz, CA, USA. The protein molecular weight was assessed by the PageRulerTM from Fermentas (Glenburnie, MD).

### LC3 and p62 Immunofluorescence staining

Histological sections of 5μm were also performed for immunofluorescence procedures of samples included in paraffin blocks. The preparation of slides was performed (deparaffinization and hydration), followed by antigen retrieval. The tissue was incubated in primary antibody anti-LC3 (M115–3) from MBL Internacional Corporation Woburn, MA, USA with a dilution of 1:500 at 4 °C overnight and after with secondary antibody conjugated with Goat Anti-Mouse IgG H&L (Cy3®) preadsorbed (ab97035) from AbCam, Cambridge, MA, USA, with a dilution of 1:500 at room temperature for 1 hour. Three fields for each sample were captured and analyzed through the Leica confocal LAS AF Lite Version 2.6 Software (Leica Microsystems, Wetzlar). All cell type stained in the cytosol for Cy3® were considered positive for quantitative analysis, which was performed by ImageJ2, by percentage of LC3 per total tissue area in a panchromatic objective field of higher magnification 40X^43^.

Indeed, we evaluated the autophagy by assessing LC3 and p62 co-localization^44^. The tissue was incubated in primary antibody anti-LC3 (M115–3 from MBL Internacional Corporation Woburn, MA, USA) and anti-p62 (ab91526 from AbCam, Cambridge, MA, USA) with a dilution of 1:500 at 4 °C overnight. The secondary antibody was Alexa Fluor® 488 (goat anti-rabbit IgG H&L: ab150077 from AbCam, Cambridge, MA, USA) in a dilution of 1:1000 at room temperature for 1 hour or Goat Anti-Mouse IgG H&L (Cy3®) preadsorbed (ab97035 from AbCam, Cambridge, MA, USA), with a dilution of 1:500 at room temperature for 1 hour. DAPI was used for nuclear staining. Three fields for each sample were captured and analyzed through the Leica confocal LAS AF Lite Version 2.6 Software (Leica Microsystems, Wetzlar). All cell type stained in the cytosol for Alexa Fluor® 488 and Cy3® were considered positive for quantitative analysis, which was performed by ImageJ2, by the percentage of co-localized pixel volume in a panchromatic objective field of higher magnification 40X^43^.

### Statistical analysis

All results were reported as means ± SEM. Data were analyzed by non-parametric Test, comparing all groups. The level of significance was set at p < 0.05.

### Ethics approval and consent to participate

This study was approved by the Ethics Committee of the University of Campinas (UNICAMP), all patient signed the informed consent form, and was performed in accordance with the Declaration of Helsinki.

### Consent for publication

Ethical approval by the ethic board of the University of Campinas (UNICAMP) and consent of patients are included in the original publications.

### Availability of data and materials

All data generated or analyzed during this study are included in this article and in the additional files.

## Electronic supplementary material

Electrophoretic gels and blots

Pouchitis Disease Activity Index (PDAI) of all patients included in the study

Ponceau-S staining of the Western blot membranes used as loading controls
