# Supplementary material for: Ileal pouch of ulcerative colitis and familial adenomatous polyposis patients exhibit modulation of autophagy markers
Source: Sci Rep. 2018 Feb 8;8:2619. doi: 10.1038/s41598-018-20938-5 (PMC5805688; doi:10.1038/s41598-018-20938-5)

Title: Ileal pouch of ulcerative colitis and familial adenomatous polyposis patients exhibit modulation of autophagy markers

Authors:

Nielce Maria Paiva, MD, MS, Lívia Bitencourt Pascoal, PhD, Leandro Minatel Vidal Negreiros, Andressa Coope, PhD, Mariana Portovedo, MS, Maria de Lourdes Setsuko Ayrizono, MD, PhD, Claudio Saddy Rodrigues Coy, MD, PhD, Marciane Milanski, PhD, Raquel Franco Leal, MD, PhD.

Supplementary Information File - Electrophoretic gels and blots

Figure Beclin 1 - Compliance with the digital image. A- Western blot analysis of Beclin-1 shown in the article (Figure 4-A). **B-** The nitrocellulose membrane of gel/blot was cropped to analyze different proteins in the same gel. **C-** Images of full-length gels and blots was included. Specific bands of Beclin 1 were labeled by a chemiluminescence reaction (SuperSignal West Pico Chemiluminescent Substrate from Pierce Biothecnology, Inc. Rockford, IL), as specified in the study methodology. The red arrow show the specific bands of Beclin -1.

A

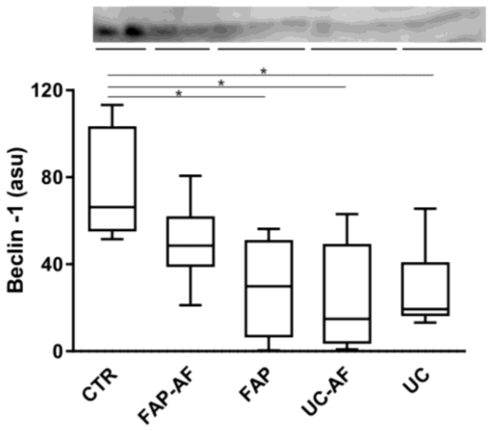

B

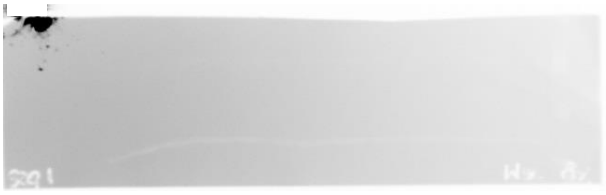

C

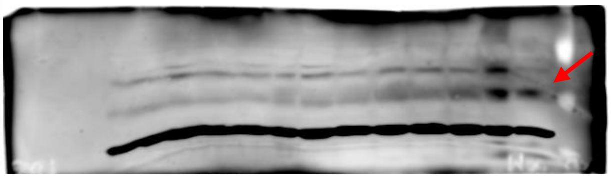

Figure LC3 II - Compliance with the digital image. A- Western blot analysis of LC3 II shown in the article (Figure 4-B). **B-** Images of full-length nitrocellulose membrane was included. **C-** Image of full-length gel and blot was included. Specific bands of LC3 II were labeled by a chemiluminescence reaction (SuperSignal West Pico Chemiluminescent Substrate from Pierce Biothecnology, Inc. Rockford, IL), as specified in the study methodology. The red arrow show the specific bands of LC3 II.

A

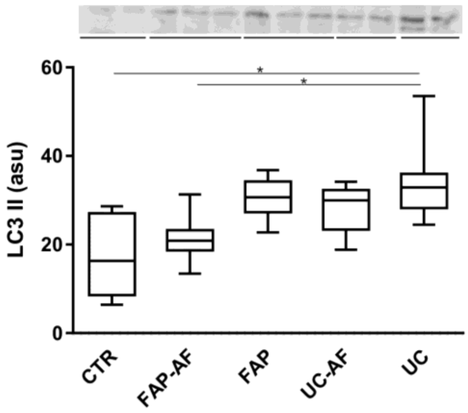

B

C

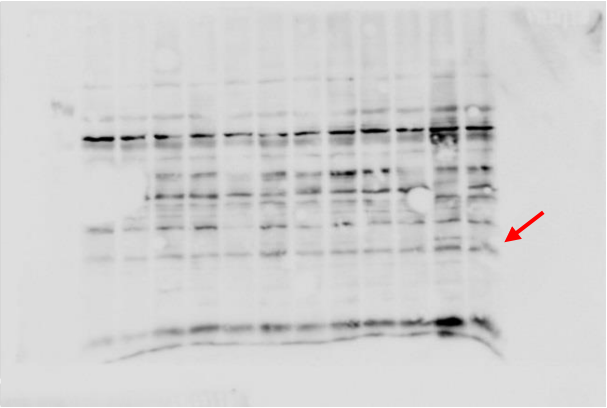

Figure P62 - Compliance with the digital image. A- Western blot analysis of P62 shown in the article (Figure 4-C). **B-** The nitrocellulose membrane of gel/blot was cropped to analyze different proteins in the same gel. **C-** Image of full-length gel and blot was included. Specific bands of P62 were labeled by a chemiluminescence reaction (SuperSignal West Pico Chemiluminescent Substrate from Pierce Biothecnology, Inc. Rockford, IL), as specified in the study methodology. The red arrow show the specific bands of P62.

A

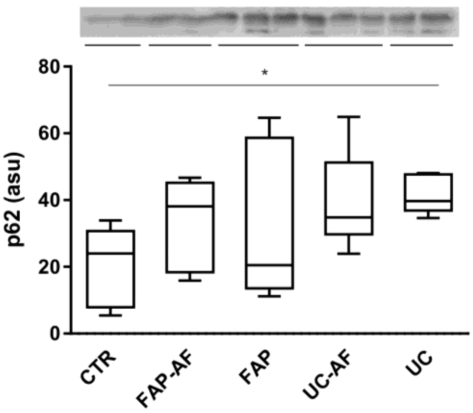

B

C

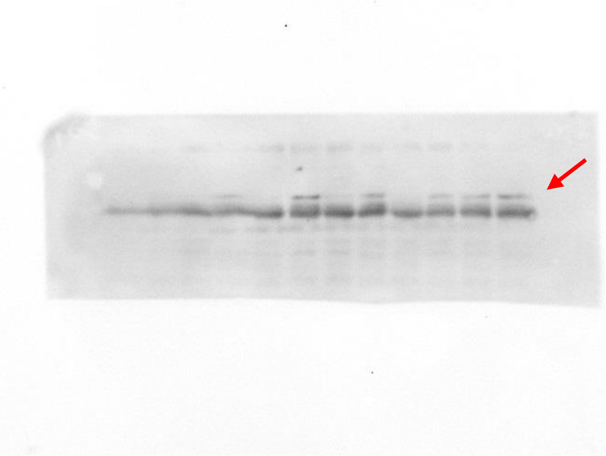

Figure HSC70 - Compliance with the digital image. A- Western blot analysis of HSC70 shown in the article (Figure 4-D). **B-** The nitrocellulose membrane of gel/blot was cropped to analyze different proteins in the same gel. **C-** Image of full-length gel and blot was included. Specific bands of HSC70 were labeled by a chemiluminescence reaction (SuperSignal West Pico Chemiluminescent Substrate from Pierce Biothecnology, Inc. Rockford, IL), as specified in the study methodology. The red arrow show the specific bands of HSC70.

A

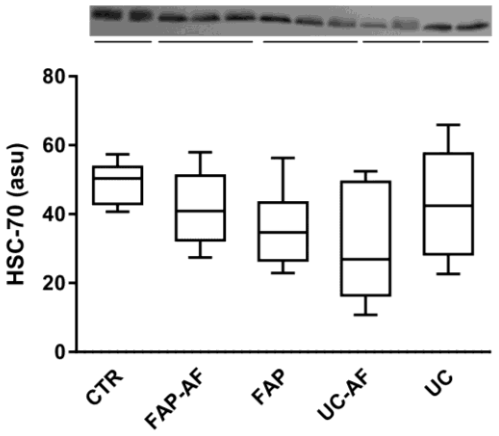

B

C

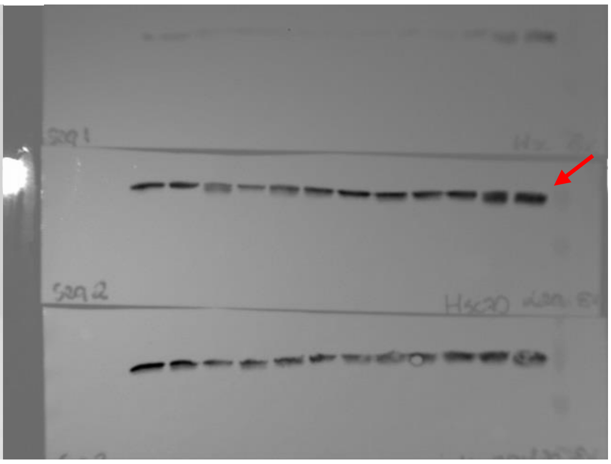

Supplement: Supplementary file 1 — Electrophoretic gels and blots [file 41598_2018_20938_MOESM1_ESM.pdf]
