# Supplementary material for: Ileal pouch of ulcerative colitis and familial adenomatous polyposis patients exhibit modulation of autophagy markers
Source: Sci Rep. 2018 Feb 8;8:2619. doi: 10.1038/s41598-018-20938-5 (PMC5805688; doi:10.1038/s41598-018-20938-5)
Supplement: Supplementary file 2 — Pouchitis Disease Activity Index (PDAI) of all patients included in the study [file 41598_2018_20938_MOESM2_ESM.pdf]

Ileal pouch of ulcerative colitis and familial adenomatous polyposis patients exhibit modulation of autophagy markers

Authors: Nielce Maria Paiva, Lívia Bitencourt Pascoal, Leandro Minatel Vidal Negreiros, Mariana Portovedo, Andressa Coope, Maria de Lourdes Setsuko Ayrizono, Claudio Saddy Rodrigues Coy, Marciane Milanski, Raquel Franco Leal.

Supplementary Information - Pouchitis Disease Activity Index (PDAI) of all patients included in the study. Pouchitis is defined by PDAI > 7 points. (PMN=polymorphonuclear; FAP=familial adenomatous polyposis; UC=ulcerative colitis)

Elements of the Pouchitis Disease Activity Index																		
Clinical Conditions	Criteria	FAP patients									UC patients							
		Score	Patient 1	Patient 2	Patient 3	Patient 4	Patient 5	Patient 6	Patient 7	Patient 8	Patient 1	Patient 2	Patient 3	Patient 4	Patient 5	Patient 6	Patient 7	Patient 8
Stool frequency	Usual postoperative stool frequency	0	0	0	0	0	0	0	0	0	0	0	0	0	0	0	0	0
	1-2 stools/day > postoperative usual	1	0	0	0	0	0	0	0	0	0	0	0	0	0	0	0	0
	3 or more stools/day > postoperative usual	2	0	0	0	0	2	0	0	0	0	0	0	0	0	0	0	0
Rectal bleeding	None or rare	0	0	0	0	0	0	0	0	0	0	0	0	0	0	0	0	0
	Present daily	1	0	0	0	0	0	0	0	0	0	0	0	0	0	0	0	0
Fecal urgency or abdominal cramps	None	0	0	0	0	0	0	0	0	0	0	0	0	0	0	0	0	0
	Ocasional	1	0	0	0	0	0	0	0	0	0	0	0	0	0	0	0	0
	Usual	2	0	0	0	0	0	0	0	0	0	0	0	0	0	0	0	0
Fever	Absent	0	0	0	0	0	0	0	0	0	0	0	0	0	0	0	0	0
	Present	1	0	0	0	0	0	0	0	0	0	0	0	0	0	0	0	0
Endoscopic inflammation	Edema	1	0	0	0	0	0	0	0	0	0	0	0	0	0	0	0	0
	Granularity	1	0	0	0	0	0	0	0	0	0	0	0	0	0	0	0	0
	Friability	1	0	0	0	0	0	0	0	0	0	0	0	0	0	0	0	0
	Loss of vascular pattern	1	0	0	0	0	0	0	0	0	0	0	0	0	0	0	0	0
	Mucous exudate	1	0	0	0	0	0	0	0	0	0	0	0	0	0	0	0	0
	Ulceration	1	0	0	0	0	0	0	0	0	0	0	0	0	0	0	0	0
Acute histologic inflammation	Mild PMN infiltration	1	1	1	1	1	1	0	0	1	1	0	1	2	1	0	0	0
	Moderate PMN infiltration + crypt abscess	2	0	0	0	0	0	0	0	0	0	0	0	0	0	0	0	0
	Severe PMN infiltration + crypt abscess	3	0	0	0	0	0	0	0	0	0	0	0	0	0	0	0	0
	Ulceration per low-power field <25%	1	0	0	0	0	0	0	0	0	0	0	0	0	0	0	0	0
	Ulceration per low-power field 25 to 50%	2	0	0	0	0	0	0	0	0	0	0	0	0	0	0	0	0
	Ulceration per low-power field >50%	3	0	0	0	0	0	0	0	0	0	0	0	0	0	0	0	0
Pouchitis Disease Activity Index			1	1	1	1	3	0	0	1	1	0	1	2	1	0	0	0
