# Supplementary material for: Ileal pouch of ulcerative colitis and familial adenomatous polyposis patients exhibit modulation of autophagy markers
Source: Sci Rep. 2018 Feb 8;8:2619. doi: 10.1038/s41598-018-20938-5 (PMC5805688; doi:10.1038/s41598-018-20938-5)

Ileal pouch of ulcerative colitis and familial adenomatous polyposis patients exhibit modulation of autophagy markers

Authors: Nielce Maria Paiva, Lívia Bitencourt Pascoal, Leandro Minatel Vidal Negreiros, Mariana Portovedo, Andressa Coope, Maria de Lourdes Setsuko Ayrizono, Claudio Saddy Rodrigues Coy, Marciane Milanski, Raquel Franco Leal.

Supplementary Figure - Ponceau-S staining of the Western blot membranes used as loading controls. Total protein staining confirmed equal loading in Western blot analysis of Beclin-1, LC3, p62 and HSC-70 expressions in intestinal mucosa (A, B, C) as presented in Figure 4.

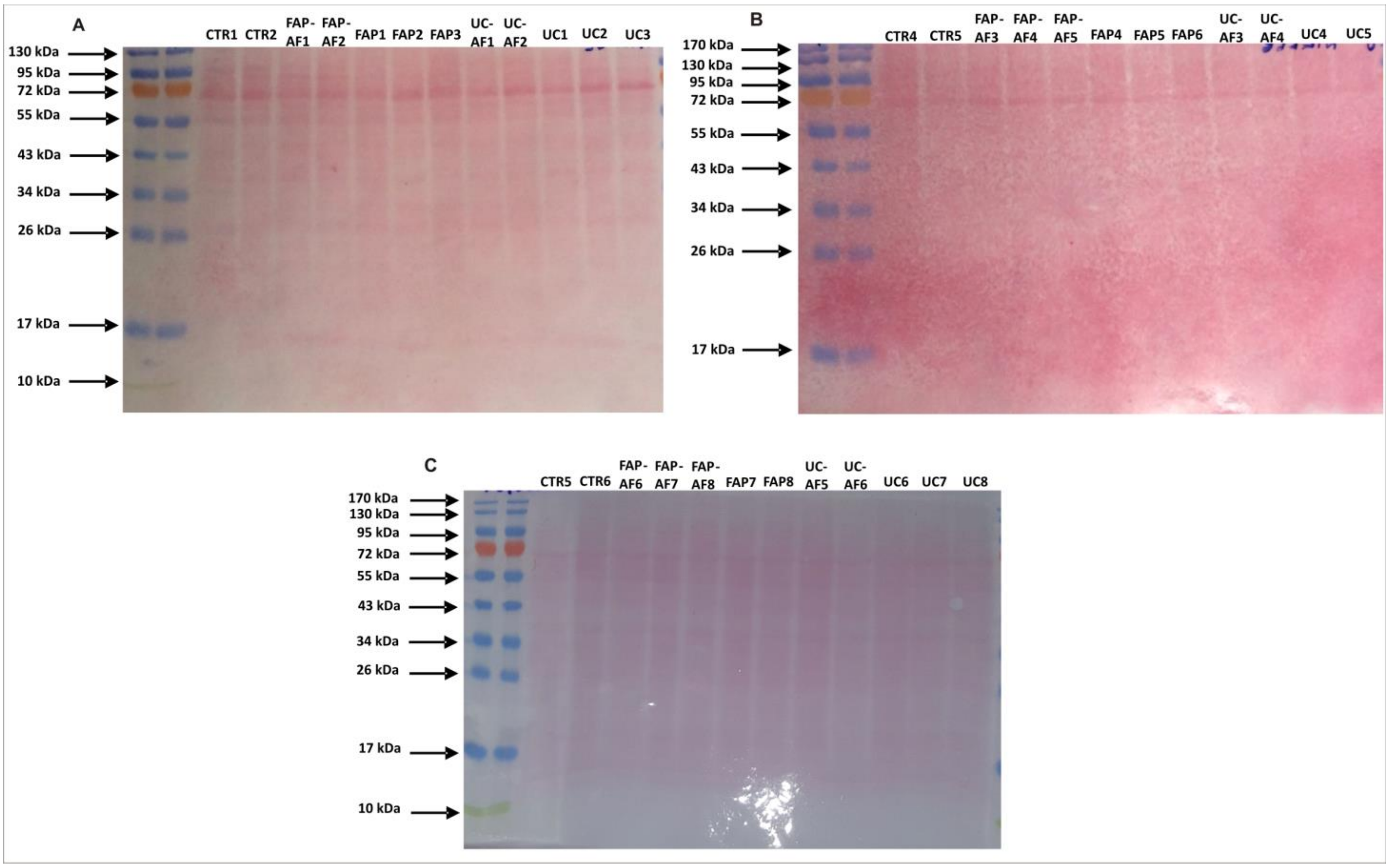

Supplement: Supplementary file 3 — Ponceau-S staining of the Western blot membranes used as loading controls [file 41598_2018_20938_MOESM3_ESM.pdf]
